# Usability of Standards for Scaffolding in a Health Sciences Programme: A feasibility Study

**DOI:** 10.1186/s12912-024-01975-0

**Published:** 2024-05-07

**Authors:** Beloved Masava, Champion N. Nyoni, Yvonne Botma

**Affiliations:** School of Nursing, University of the Free State, P.O. Box 339, 9300 Bloemfontein, South Africa

**Keywords:** Feasibility, Health Sciences Programme, Nursing, Pilot study, Scaffolding, Standards, Usability

## Abstract

**Background:**

Standards contribute to comprehensive and programmatic implementation of educational strategies, such as scaffolding. Although the development of educational standards follows a rigorous consensus approach, they are socially constructed and could result in varied interpretations by users. Reports of varied implementation of standards in health professions education underscore the need to test the developed standards for scaffolding in health sciences programmes. Usability entails determining whether a product like standards works as intended under the expected conditions and contexts. This study aimed to describe the usability of standards for scaffolding in a health sciences programme through a pilot study.

**Methods:**

A multi-method design employing user and expert-based usability evaluation techniques sought to describe the usability of the standards for scaffolding in a three-year pre-registration nursing programme. The user sample of nurse educators drawn from the programme, conducted a self-assessment on scaffolding practices in the programme using a developed standards checklist. For the expert sample, three-panel members with an understanding of the discipline and programme context were purposively sampled. These panelists studied the users’ self-assessment reports before completing an author-generated heuristics checklist to support or refute any of the standards. Descriptive statistics, comparative and content analysis were applied to analyse data from users’ interviews and expert’s completed heuristics checklist, determining the standards’ usability, and identifying the usability flaws or strengths.

**Results:**

The users had three or more years of teaching experience in the competency-based curriculum for nursing. The experts shared an average of 16 years of experience in teaching in higher education, and seven years of experience in quality assurance and programme accreditation. The four standards had a usability score of above average (68%). Seven usability strengths and four usability flaws were identified. Usability flaws related to misinterpretation of some criteria statements and terminologies, multiple meanings, and users’ challenges in generating evidence for some criteria.

**Conclusions:**

The pilot study revealed the context-based ‘truth’ regarding the fidelity of a health sciences programme evaluation on scaffolding, as well as identifying the ideal contextual conditions in which the standards for scaffolding health sciences programmes would work best. The identified usability flaws highlighted the need for further revisions of the standards. Future research on the feasibility of the standards in other health sciences programmes and contexts is recommended.

**Supplementary Information:**

The online version contains supplementary material available at 10.1186/s12912-024-01975-0.

## Background

Even though global standards for health care education and practice are a product of prescribed rigorous processes with input from field experts [1, 2], they are not immune to technical ambiguity and inconsistent interpretations [3, 4]. Grant and Grant [5] (2022) argue that these multiple interpretations may be inevitable given standards result from social construction processes relative to the underlying pedagogical or political context. The potential for technical ambiguities and varied interpretations of standards in different settings underscores the need to consider the usability of global innovations within the context of a health sciences programme in which they are intended to operate [5, 6]. The availability of resources, underpinning philosophies, and the political, social, environmental, and organisational culture, constitute internal and external contextual determinants that influence the translation and interpretation of global educational innovations such as standards [7–10].

Educational standards are the currency for quality assurance in health sciences programmes [11, 12]. They are essential in improving the consistency, efficiency, and quality of education for health professionals [11, 13–19]. The quality of health sciences education is enhanced through the application of standards in areas such as curriculum development [19, 20], simulation-based education [21, 22], students assessment [2, 23], programme evaluations and accreditation [11, 14, 17, 23, 24]. The standards to direct scaffolding practices in health sciences programmes were developed through a global consensus approach in an earlier stage of the larger study [25]. These standards need to be tested for contextual fidelity and practical application.

Scaffolding refers to the temporary support provided to assist students in constructing knowledge that is beyond their existing knowledge, ability, and experience [26–28]. The development of scaffolding theory was influenced by Vygotsky’s work on sociocultural and the Zone of Proximal Development (ZPD) theories [29]. The sociocultural learning theory postulates the value of students’ engagement in social interactions with the environment, peers, and educators during their construction of knowledge [29]. Vygotsky (1978), defined the ZPD as ‘the distance between the student’s actual abilities, as determined by his/her independent task performance abilities, and the student’s potential development, as determined by his/her task performance under the guidance of an educator or knowledgeable peer’ [29]. Vygotsky [29] believed that a student can learn any complex knowledge or skill during learning interactions given relevant ‘scaffolds’ are implemented through the student’s related zone of proximal development. Wood, Bruner and Ross (1976), further advanced the notion of scaffolding, describing it as tailored support provided to students by educators based on the students’ current expertise and should suit their potential performances [30]. Therefore, scaffolding becomes an important educator’s tool given its potential to progress students from their current knowledge state to potential performances on knowledge discovery and complex problem-solving abilities. Without scaffolding, it would be difficult for students to complete the complex tasks and assignments characterising health sciences programmes [31].

Scaffolding is essential in assisting health sciences students to effectively acquire professional and context-specific competencies [32, 33]. However, the application of scaffolding in health sciences programmes is fragmented, with evidence of its application in silos at the modular level and on specific learning platforms [32], underscoring the need for standards [25]. Global educational standards, including those relating to scaffolding, are practical if applied and modified within the context of a health sciences programme [5], underscoring the need for usability testing.

Usability refers to the extent to which standards for scaffolding can be utilised by its intended users in the diverse settings and learning platforms of health sciences programmes to support learning ‘effectively, efficiently, and satisfactorily’ [34, 35]. Usability is operationalised as the feasibility, effectiveness, adaptability, applicability, credibility, clarity, and relevance of standards to the scaffolding practices of both the local and global health sciences programmes [16, 36–38]. The assessment of usability is accomplished through expert and or user-based evaluation [39–41]. When applied concurrently, expert and user-based evaluation contribute to complementary findings valuable to determine holistic perspectives on the degree of usability of a product [42]. Usability testing using established heuristics is usually applied in the performance evaluation of electronic-based healthcare practice artefacts, ensuring the accuracy of diagnostic tests and the efficiency of computer-based systems for patient treatments [39, 41, 43, 44]. However, studies have operationalised usability to include the feasibility evaluation of non-technological tools and standards [45]; and the application of author-generated heuristics to evaluate mobile health programs [43]. Studies further recommend usability testing and ‘realist evaluation’ of artefacts such as standards to improve future use [5, 34].

The myriad of contexts and settings under which health sciences programmes operate matters when adopting global educational innovations such as standards [5, 6]. Standards for scaffolding in health sciences programmes referred to in this study were socially constructed using a blend of literature review findings, and assumptions or opinions of international experts [25]. Hence, the standards may not represent the ‘global truth’ of scaffolding that matches all the contexts in which health sciences programmes operate [5]. Furthermore, Bias [46] urges the designing and testing of innovations to ‘fit the purpose of the user and the underlying context’. These affirmations underscore the need for a ‘realist evaluation’, to determine the usability of standards in the context of a nursing programme [5]. The recently developed standards for scaffolding in health sciences programmes [25] are yet to be subjected to such an evaluation either in low-, middle- or high-income countries. The pilot of these standards for scaffolding in the local context of a health sciences programme further highlights the authors acknowledgement of the prospect of varied interpretations of global educational policies [5]. Therefore this article describes the usability of standards for scaffolding in a health sciences programme with anticipation to reveal the context-based ‘truth’ regarding the fidelity of application of such standards in evaluating scaffolding practices among health sciences programmes.

## Methods

**Fig. 1:**
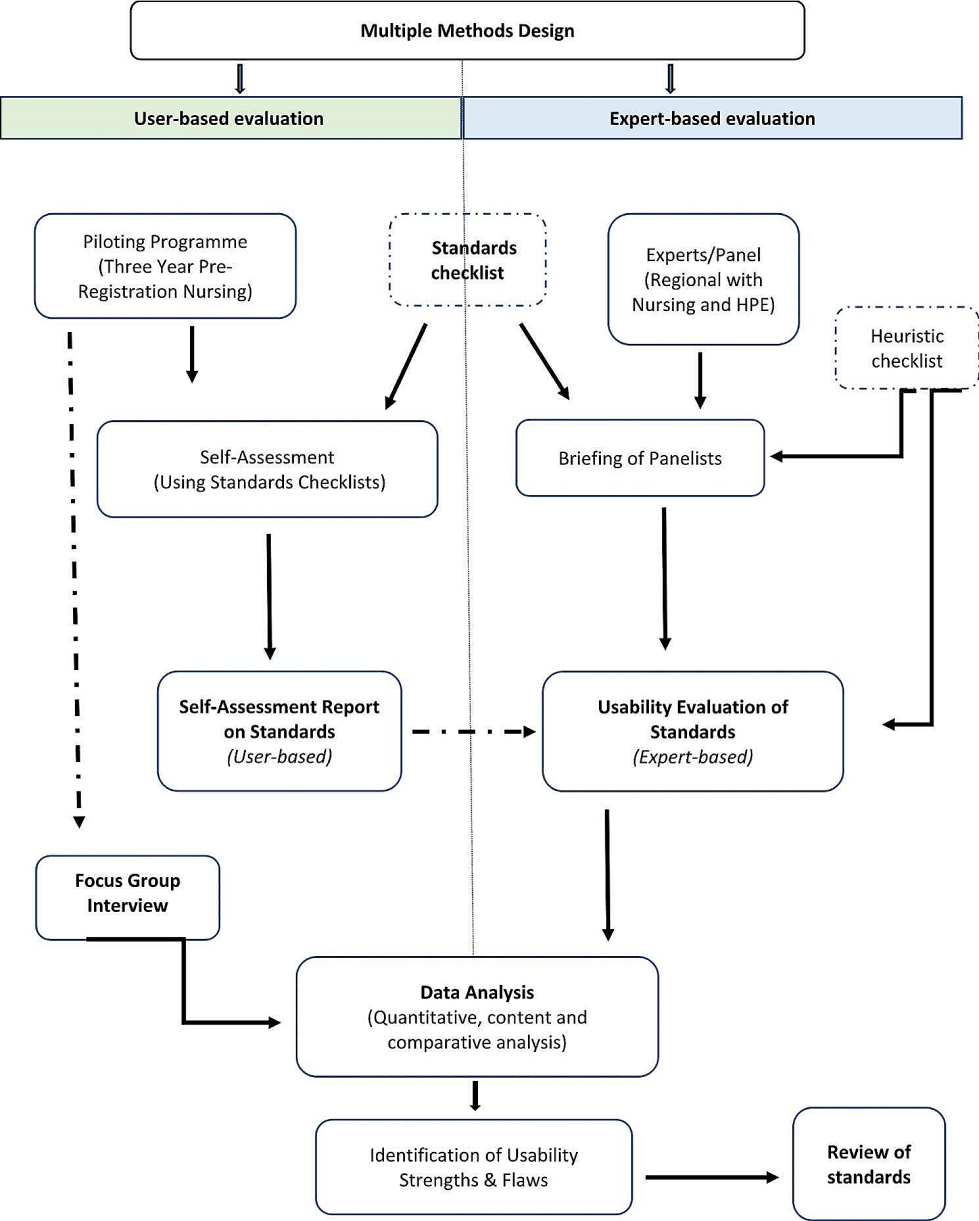
Flow chart of standards pilot methods applied (author generated)

As shown in Fig. 1, a multi-method design comprising user and expert-based usability evaluation techniques [44, 47] was applied to describe the usability of the standards for scaffolding in a nursing programme. In the initial phase, users applied the developed standards’ checklist to conduct a self-assessment on scaffolding practices in the nursing programme, before participating in a group interview. During the second phase, experts examined the users’ self-assessment report to respond to the heuristics of the standards on how the users’ interpreted, applied, and adapted the standards for scaffolding in the programme. The investigators then triangulated the data obtained from both phases to draw inferences on the degree of standards usability, identifying usability strengths and flaws.

## Selection of the health sciences education programme

Standards for scaffolding were pilot tested in a three-year pre-registration nursing programme that is accredited by the local professional and higher education regulatory bodies. The programme was selected due to its convenience and accessibility to the investigators. Social constructivist, student-centred, and competency-based approaches guide the teaching and learning in the programme [48]. The competency-based curriculum is underpinned by principles of constructivism, constructive alignment, authenticity, and scaffolding [48]. These principles and the curriculum demand from students to construct knowledge using cooperative learning strategies, study guides, and support from facilitators to achieve stated profession-specific competencies. The espoused context-related competencies, derived through situational analysis and stakeholder involvement [49], seek to prepare the graduates of the programme to match the country’s health needs [50, 51]. Furthermore, the design of the programme emphasises scaffolding during learning on various platforms, including traditional classrooms, virtual spaces, simulation laboratories, the community, and primary health care and hospital clinical settings [52, 53].

### Sampling of users

Purposive sampling was followed to select at least three nurse educators with a rich understanding of the nursing curriculum, teaching and learning experiences. The Head of the Programme, together with three programme representatives with the most experience and expertise in teaching and quality assurance self-evaluations agreed to participate in the study.

### Sampling and recruitment of experts

Five experts were recruited in consultation with the country’s Council on Higher Education. Purposive sampling of the experts was followed to increase the chances of obtaining experienced people with an understanding of the context of the programme and discipline to guarantee rich inputs regarding the usability of standards. Experts with qualifications in quality assurance in higher education and a minimum of a master’s degree in a health science programme were selected. Also, a minimum of two years working experience in accreditation was a needed requirement for selection. Eligible experts currently facilitating the programme were excluded from the study.

The five eligible panelists received official invitations and an information leaflet via electronic mail. The information leaflet detailed the study’s data collection processes and the roles of the panelists. In addition, the panelists indicated their willingness to participate via electronic mailings.

### Data collection

Data collection was managed in two phases; namely the user-based and expert-based evaluation methods.

### The user-based standards evaluation process

The first author briefed the users representing the programme, regarding the roles, expectations and processes involved in piloting the standards. The evaluation required the users to apply the provided standards checklist to carry out a self-evaluation of scaffolding practices in the programme.

The four standards related to scaffolding developed through a Delphi consensus approach reported elsewhere [25] were presented as a checklist in a Microsoft Word format. The checklist presented the standards and verification criteria, together with a column to capture the summary of findings and evidence for the existence of the standards.

The users read through each of the standards and reflected on the performance of the programme on the standards before submitting a self-assessment report to the investigators. The report summarised the users’ perceived performance of the programme on each of the standards, and cited sources of evidence to justify their judgements. Hence, the self-assessment report yielded data related to how the users applied the standards for scaffolding.

The first author conducted a group interview to debrief the users upon completion of the self-assessment. The debriefing was conducted virtually using a semi-structured interview guide developed by the authors for this study. See supplementary file for Faculty Interview Guide. The interview guide was pilot tested with two facilitators in the midwifery programme. Audio recording and field notes captured the representatives’ experiences with the use of the standards.

### Expert-based standards evaluations

The three experts who confirmed their willingness to participate received orientation on the standards, related heuristics and their roles in the data collection processes. The panel accessed the programme’s self-assessment report and related heuristics checklist. See Heuristic Checklist in Addendum.

An author-generated heuristic checklist consisting of five heuristics formulated based on a synthesis of usability principles from the literature [2, 19, 45]. These principles relate to clarity, appropriate terminology use, matching of self-assessment evidence, utility for health sciences and effectiveness in reflecting scaffolding. The heuristics were also formulated to evaluate if the developed standards conformed with the principles of standards presentations [2, 19, 45]. The checklist required the experts to agree or disagree on the usability of each standard. Space for additional comments and/or suggestions for restructuring the standards was provided. The heuristics checklist was configured into the REDCap® application, and the resultant link was shared for easy access and completion by the experts.

Before completing the heuristics checklist, panellists read through the programme self-assessment report to understand how the programme users interpreted, applied, and adapted the standards for scaffolding to the programme context. Furthermore, the panel critically examined the appropriateness and relevance of the evidence presented by the programme on each of the standards. After studying the users’ self-assessment report, experts independently proceeded to complete an author-generated heuristics checklist to support or refute any of the standards/criteria. The heuristics questions guided the experts to identify any standards’ usability strengths or flaws, including issues with structuring or wording [44, 54]. The experts were given a minimum of two weeks to submit the completed heuristics checklist and were reminded via email two days before and two days after the deadline.

### Data analysis

The first author downloaded the expert evaluators’ completed heuristics checklists from REDCap®, and checked them for completeness before commencing with data analysis. Data analysis involved identifying the standards for scaffolding that were deemed to have either conformed or not conformed to any of the usability principles on the checklist [41]. Comparative and content analysis were applied to identify usability flaws from the checklists completed by the three experts, triangulating with data from the interviews with the users.

Descriptive statistics were applied to describe usability problems and the degree of conformity or non-conformity of the standards in terms of the stated heuristics. The expert responses on grading the usability were captured either as a Yes, No or Maybe for each of the heuristics (Yes = 2; Maybe = 1; No = 0) [55]. Each standard was examined against five heuristics with a potential overall score of thirty. A compliance score of each standard per heuristic was obtained by adding the weighted scores of the experts’ responses. The resulting scores were added together to determine the total usability score of every standard and each total score was then multiplied by $$3{\raise0.5ex\hbox{$\scriptstyle 1$}\kern-0.1em/\kern-0.15em\lower0.25ex\hbox{$\scriptstyle 3$}}$$ to convert the original scores of 0–30 to 0-100. A standard was interpreted as having a usability problem when its tally score was less than twenty or 68% [56]. Therefore, fractions and percentages were used to describe the conformity of the standards to usability principles.

Data obtained from the interviews were transcribed verbatim and analysed deductively in line with standards heuristics. Potential standards usability strengths and flaws emanating from the interviews were identified. The themes emerging from this data were corroborated with findings from the expert-based heuristic evaluation. The identified usability issues were collated to establish a list of usability findings and were presented in a table format to account for various problems identified by the panel and users. Through a virtual meeting, the investigators discussed and agreed on the finalised list of standards usability strengths and flaws.

### Ethics issues

This study was conducted in accordance with the key ethical principles of the Declaration of Helsinki (2013 version) and the South Africa National Health Research Ethics Council (NHREC) report of 2015. Moreover, this study was approved by the Health Sciences Research Ethics Committee of the University of the Free State (UFS-HSD2020/1864/2302) and the National Health Research Ethics Committee (NH-REC) of the Kingdom of Lesotho (ID 118–2020). Written permission to pilot scaffolding standards was sought from the institutions’ respective gatekeepers. The programme representatives conducted the review of the scaffolding standards in the absence of the investigators, to prevent undue influence of academic, professional, and relational status to interfere with the usability judgements. We applied a set of criteria to identify experienced people with an understanding of the context of the programme and discipline to form the expert panel. Participants received explanations regarding the purpose of the study prior to giving informed consent to participate in the study. Confidentiality was maintained by replacing identifying information of experts with codes prior to data analysis.

## Results

A synthesis of the data from both the users and the experts shed light on the number and nature of the usability problems, and the strengths that were identified.

### Participants demographics

One panel member was from a local nursing college, while the other two were from universities in Sub-Saharan Africa. At the time of data collection, all experts worked in a higher education institution (HEI). All three experts had a minimum of a PhD with a specialisation in nursing education. One expert had obtained an additional health professions education (HPE) qualification. Between them, the experts shared an average of 16 years of experience in teaching in higher education and seven years of experience in quality assurance and programme accreditation.

### Usability scores

Table 1 shows that the four standards scored above average of twenty or 68 percentile [56, 57]. Standard 4 had the highest usability score of 27/30, and standard 2 had the lowest with 22/30.

**Table 1:** Results from heuristic evaluation

Heuristics	Total rating per standard compliance to usability principles			
	Standard 1	Standard 2	Standard 3	Standard 4
**Heuristic 1**: The standard and accompanying criteria are clear.	4	4	5	4
**Heuristic 2**: The standard and accompanying criteria are phrased using appropriate terminology.	5	3	5	6
**Heuristic 3**: The self-assessment findings are supported by evidence that matches the standard/criteria requirements.	6	5	5	5
**Heuristic 4**: The standard and accompanying criteria are useful for health sciences programmes.	6	5	6	6
**Heuristic 5**: The standard or accompanying criteria are effective in evaluating scaffolding.	4	5	5	6
**Scores out of 30**	**25**	**22**	**26**	**27**

### Usability strengths

The data from the expert-based evaluations and user interviews revealed seven usability strengths of the standards. The identified strengths were mapped to the stated heuristics principles and linked to related data sources (Table 2).

**Table 2:** Identified usability strengths

Usability strength described	Source of data	Heuristic supported
1. An exciting and enriching experience	**User 1**: “The experience was intriguing”. **User 2**: “Exciting and enriching…. Open eyes on scaffolding needs, a learning experience. It gave me a deeper understanding …”.	**Heuristic 4**: The standard or accompanying criteria are useful for health sciences programmes.
2. Limitations on programme scaffolding practices identified	**User 2**: ”…. Aid to identify some of the gaps in scaffolding practices in the program that we did not know exist.” **Expert 1**: “Accreditation reports and external moderation reports could have helped in verifying some of the standards”. **Expert 3**: “The human resource aspect is not expanded. Who are the human resources in this case, and what are the skills and knowledge of these human resources regarding scaffolding?”	**Heuristic 5**: The standard or accompanying criteria are effective in evaluating scaffolding.
3. Reinforced existing programme scaffolding activities	**User 1**: “It reinforced on scaffolding principles demanded in programme ….” **Expert 2**: “There is clear evidence of student support during learning”.	**Heuristic 5**: The standard or accompanying criteria are effective in evaluating scaffolding.
4. Critical examination of the programme scaffolding intentions	**User 2**: “We experience interrogation of the program from curricular mapping to classroom activities, dissecting a program to the required level”. **Expert 2**: “While this section ………. is too long, it addresses the different types of resources adequately”.	**Heuristic 5**: The standard or accompanying criteria are effective in evaluating scaffolding. **Heuristic 3**: The self-assessment findings are supported by evidence that matches the standard/criteria requirements.
5. Provided a focused and programmatic approach to scaffolding	**User 1**: “I felt it was a focused approach to look at the programme …. across all levels. And it gave me a different understanding of the programme, especially things we took for granted on scaffolding”. **Expert 2**: “The standard and criteria are effective in evaluating the application of scaffolding at the various levels”.	**Heuristic 4**: The standard and accompanying criteria are useful for health sciences programmes. **Heuristic 5**: The standard or accompanying criteria are effective in evaluating scaffolding.
6. The standards were clear and usable	**User 1**: “They were usable, and most were straight to the point … unambiguous…”. **User 3**: “ …. usable tool that can benefit programmes….” **Expert 2**: “Although the standards and criteria can be generalised to most teaching and learning areas, it is useful to health sciences programmes”. **Expert 3**: “The programme findings provide evidence that meets requirements”.	**Heuristic 1**: The standard and accompanying criteria are clearly written. **Heuristic 4**: The standard and accompanying criteria are useful for health sciences programmes.
7. The standards demanded teamwork and collaboration for completion	**User 3**: “…us sitting together and discussing, bound your thoughts and assisted interpreting on the other criteria”. **User 2**: “Sitting together was better to remind each other … require teamwork not one-person show, where one person sits and do the evaluation …”	**Heuristic 5**: The standard or accompanying criteria are effective in evaluating scaffolding.

Users stated that the standards’ piloting exercise was exciting and enriching. They felt the experience enlightened them on the existing scaffolding practices and cultures within the programme. This opportunity to critically examine own scaffolding practices was desirable considering the scaffolding requirements stipulated in the described competency-based curriculum of the programme.

The users also verbalised that using the standards to evaluate the programme scaffolding was eye-opening as it revealed several areas often taken for granted in scaffolding learning. Likewise, the experts reported lack of evidence that could have been cited by users if certain scaffolding practices were in existence in the programme (Table 2). Meaning the standards aided in the identification of gaps related to scaffolding within the programme. Hence, the standards were valuable and applicable to offer necessary guidance on how to scaffold modules, teaching and learning and assessments in the programme.

As shown on Table 2, the users felt that the standards reinforced existing scaffolding practices in the programme, an observation which was supported by experts. Evidence on meticulous design of modules to support deep learning, references of resources, tools and models to support students’ cognitive development and clinical reasoning supported the feasibility of the standards for scaffolding. Therefore, the standards appraised scaffolding efforts by educators in the programme, a critical observation to support their usability.

The data demonstrate that the users valued the standards’ guidance to nurture an evidence-based, focused approach to scaffolding in the entire programme. They verbalised that the approach promoted a holistic view of scaffolding application that includes various knowledge disciplines and learning platforms. The observation was supported by experts who claimed that the standards and criteria were effective in evaluating the application of scaffolding at the various levels. See Table 2 for more information from the users and experts. These admissions by the users reveal that scaffolding was easily glossed over without critical consideration on the how, when and where it should be applied to support students’ competency development.

The standards and accompanying criteria were practical to support the current scaffolding practices. The users attested to have benefited from applying the standards in identifying areas needed to strengthen student support. Furthermore, the standards were useful as they assisted in critical examination of scaffolding and demanded evidence to demonstrate the attainment of deliberate practices to support learning in the programme. The experts commented on the comprehensive evidence of resources that support students learning in the programme, further attesting the value of the standards. Relatedly, the users admitted that although the standards demanded intense reflective thinking, they also promoted teamwork, and collaboration during gathering of relevant evidence to support the existence of scaffolding across various levels and modules. The views of the users were echoed by the experts, who felt that the evidence from the programme self-evaluation demonstrated that the standards were important, relevant and somehow clear to follow and could be translated to most teaching and learning areas of the health sciences programmes. The experts also reiterated that most of the standards’ areas and criteria were easy to follow and met the expectations of appraising the scaffolding requirements in the programme. See Table 2 for more details from the users and experts.

### Usability flaws

Four usability flaws were identified from the analysis of the users’ interviews and the expert evaluations. These identified flaws were mapped to the stated heuristic principles as well as the related data sources (Table 3).

**Table 3:** Usability flaws identified

Usability problem described	Source of data	Heuristic violated
1. Insufficient evidence in Standard 2: *Resources and tools for scaffolding learning*	**User 2**: “ … the other bit of a challenge was thinking through the evidence that is needed ….”. **Expert 1**: “Accreditation reports and external moderation reports could have helped in verifying some of the standards”. **Expert 3**: “The human resource aspect is not expanded. Who are the human resources in this case, and what are the skills and knowledge of these human resources regarding scaffolding?”	**Heuristic 3**: The self-assessment findings are supported by evidence that matches the standard/criteria requirements
2. Difficulty interpreting terms such as zone of proximal development (ZPD), resources, macro-, meso-, micro-scaffolding, and social and health systems science	**User 1**: ”…. And also that one that talks about the zone of proximal development ….”. **Expert 2**: “Micro-scaffolding is an interactive process between the educator and the student, however, there is no mention of the student as participant in own learning”. **Expert 3**: “Exit behaviour can be replaced by competency or exit learning outcome”.	**Heuristic 2**: The standard and accompanying criteria are phrased using appropriate health sciences terminology
3. Multiple interpretations and misinterpretations made by users on *Standards 1 and 2*	**User 3**: “It also talks of health systems …”. …although working through the macro part was a little bit also challenging…. You needed to know how to separate macro- and meso- … sometimes meso- and micro…”. **Expert 2**: “Explicit, however, some areas ……. of human resources that are programme specific and not only mention those that are not programme specific”.	**Heuristic 1**: The standard and accompanying criteria are clearly written.
4. Challenges in comprehending criteria *1.8: Modules and study guides allow multiple opportunities for ongoing assessments that help identify strengths and align the level of support and guidance to the individual student’s learning needs and context complexity*	**User 2**: “ … there was one which was a bit too long and …now you had to think and rethink what this criterion is asking for ….” **User 2**: “ … first standard criteria was lengthy and complex to comprehend”.	**Heuristic 1**: The standard and accompanying criteria are clearly written.

The data revealed that there was insufficient evidence available to support scaffolding practices in standard 2. Users felt challenged to generate evidence to support this standard, which focused on resources and tools for scaffolding learning. The experts echoed the observation and suggested additional evidence to cover the gap. There were also reports relating to the difficulty experienced by users when interpreting some terms used in phrasing the standards and criteria. The zone of proximal development, macro-, meso-, micro-scaffolding and systems sciences were examples of terms cited. The use of such terms without a glossary list could result in standards misinterpretations. Also, some multiple interpretations and misinterpretations of standards by users were reportedly caused by ambiguity in some of the criteria related to standards one and two. Reports from users also cited criterion 1.8 as bulky and difficult to comprehend. Experts warned that such a long criterion could lose readers and may also result in multiple interpretations.

## Discussion

Global standards need to be evaluated within the context in which they are intended to operate to assist in identifying usability flaws and ultimately improving standards applicability. This article outlined the processes and outcomes of the usability evaluation of standards for scaffolding in a health sciences programme conducted through a pilot study. Experts examined the users’ self-assessment report to respond to the heuristics of the standards on how the users’ interpreted, applied, and adapted the standards for scaffolding in the programme. The findings demonstrated that the standards were generally usable, and relevant although four flaws critical to enhance the usability and transferability of the standards were identified. The study findings could assist in refining the standards and providing evidence-based recommendations relevant to health sciences programmes in low-resource settings.

The application of a multi-method approach to evaluate the usability of the standards contributed to the value of this study in several ways. First, complementing the findings from the user- and expert-based approaches represented the views and judgements of both regarding the feasibility of the standards [40, 41]. Usability is about determining whether a product works as intended under the expected conditions [34, 58]. The users’ concern is about what would work best [42] in directing the programme scaffolding processes. Besides, the authors’ consideration of the users’ experience on the application of the standards, can be valuable in the review of the standards to enhance their acceptability and usability by the stakeholders [41]. Conversely, the selected experts provided critical views on standards usability based on their experiences of applying similar educational standards. Hence, merging the user’s experiences with expert judgements of the standards provided the necessary holistic interpretation of what works best for both the users and the experts who represent the developers. Studies that have combined the two usability evaluation methods reported similar benefits of representative and comprehensive findings that ultimately improved the usability of the related innovations [40, 44].

Furthermore, the merging of user-based and panel evaluations enhanced the rigour of this study [46]. The application of the standards by users during self-assessment provided an opportunity for a ‘realist evaluation’, which was necessary to establish the context-based ‘truth’ of the fidelity of using the standards in future evaluations of scaffolding in health sciences programmes [5]. The opportunity for data corroboration from the two methods provided the necessary validation for the usability of the standards [46]. Expert- and user-based evaluations and the application of quantitative and qualitative elements of data analysis ensured that usability was considered not only from the point of user satisfaction, but also included essential data on the performance of the standards as concluded by experts [44, 46].

The user representatives were positive regarding opportunities to adopt the standards to enhance scaffolding in the nursing programme as reported in this article. This finding was anticipated since this programme’s design of competency-based education demands the application of scaffolding [52] and will therefore probably be evaluated on the extent of the application of the scaffolding requirements [59]. The same drive to implement the standards for scaffolding cannot be guaranteed in other health sciences programmes, given the widely reported variation in the implementation of standards in medical education [9, 13, 18, 37, 50, 60, 61]. Besides, the standards for scaffolding in health sciences programmes are not prescriptive [25], but rather seek to improve practices focused on the academic support of students. However, De Silva and colleagues [45]warned that unless there are punitive measures against health educational standards non-implementers and organisations may continue their reluctance to implement innovations into practice [45].

Acknowledged principles of the presentation of standards distilled from the literature were applied to formulate heuristics statements in this study. These heuristic statements sought to support the experts’ decisions regarding the feasibility of the standards and the relevance of the evidence supporting the existence of each standard. Studies have testified to the value of heuristics in supporting or refuting the degree of usability of healthcare practice and educational technological innovations [43, 62, 63]. However, there is no study which has reported on the application of author-generated heuristics to test the practicality of health professions education standards. Feasibility studies have shown reliance on research designs that elicit the perceptions of the users of standards to make inferences regarding the appropriateness of local and global medical or nursing education standards [14, 45, 61]. This study borrowed ideas on using heuristics to support the judgement of experts regarding the extent of the usability of the standards [64]. The use of heuristics in this study was a robust and defensible strategy generating evidence that sought to improve the functionality of the standards for scaffolding, which align with similar conclusions by Khajouei and colleagues in a study on the usability of health information systems [41].

Several comments from the users and observations from the experts demonstrated that the standards were usable in evaluating scaffolding in a pre-registration nursing education programme. Both sets of comments confirmed that the standards were useful in identifying gaps in practice, and limitations on scaffolding, critical areas for quality improvement relative to programmatic scaffolding. In addition, the standards provide a useful comprehensive view of the application of scaffolding across various knowledge disciplines and learning platforms. The findings of this study supported the overall purpose of standards, namely to promote the uniformity of educational and quality assurance practices in health sciences education, inclusive of student support [13, 18, 19, 22]. Furthermore, both the users and the experts concurred regarding their judgement on the usability of each standard and highlighted the value of these standards in scaffolding across the entire programme. Currently, there is limited literature on scaffolding practices across programmes [65], as the approach is often applied in silos in selected modules and learning platforms [32, 65]. Richardson and colleagues [65] recommended the application of programme-centred scaffolding design to enhance the quality of learning of students in a healthcare administration education programme. Evidence of the usefulness and applicability of the standards within a health sciences programme context is applauded, as it acts as evidence of the practicality of the standards.

However, four usability flaws were identified from this pilot. The usability flaws ranged from the use of terms with the potential for multiple or misinterpretations to challenges in generating evidence when evaluating some of the standards. In 2012, the World Federation for Medical Education (WFME) [2] recommended the use of annotations of terms used in medical education standards statements to improve clarity and uniformity regarding the interpretation of such standards. Health sciences educators may not be familiar with some educational terms used in these standards which resulted from the political consensus approach of the standards development process [3]. Accordingly, in their standards documents, the WFME 2021 report [66] and the INACSL Standards Committee report [22] clarified selected concepts deemed necessary to improve comprehension and application of the related standards; thus, striving to eliminate technical ambiguity. The terms identified for further clarity in this pilot will be included in the standards for scaffolding package for future publications and use. Therefore, the pilot unearthed misconceptions and misinterpretations of the terms used in the standards statements, something which would have been impossible to achieve without it.

### Strengths and limitations

The application of both user- and expert-based usability evaluation methods and data triangulation increased the probability of identifying usability problems related to standards for scaffolding in health sciences programmes [39, 42]. The use of three experts in a heuristic evaluation was adequate and has shown commendable benefits in diagnosing at least 80% of the usability flaws from the innovation [39, 64]. The recruitment of available experts with vast experience in the application of standards, familiar with the geo-political context, facilitated the identification of potential usability flaws [44] that would have been difficult to identify by anyone else unfamiliar with the setting. Selecting and recruiting experts with a nursing education background was based on practicality as well as enriching authentic perspectives of standards usability relative to the needs of the discipline [50].

This study has a number of limitations and potential biases. The first author of this article, identifies as a PhD student, worked at the institution at the time of the pilot. The recruitment of the programme participants and organisation of the self-review processes by an independent quality assurance committee meant to prevent undue researcher influences on the study outcomes. Nevertheless, the authors in-depth understanding and inside knowledge of the topic, pilot programme, the curriculum, and contextual elements mentioned by the participants may have informed the data analysis and conclusions reached in this study. Without such inside knowledge, the authors may not have recognised some of the patterns discovered through data triangulation. Moreover, the piloting of the standards in only one of the well-established nursing programmes could have contributed to the overall success of this pilot exercise, something which may not be feasible with newer programmes. This can be explicated by the fact that the piloting institution was the first in the country to report successful implementation of the competency based curriculum [53]. Nonetheless, to date, this was the first study that has generated some useful insights into the feasibility of the standards for scaffolding in a health sciences programme. However, it is beyond the scope of this paper to fully present the various ‘scaffolds’ observed in the programme during the pilot, as such data of full scale evaluation of programmatic scaffolding is planned for reporting in a forthcoming study.

Another limitation of this study is that its findings cannot be generalised to health sciences programmes including those of the discipline of nursing. Various health sciences programmes operate in different contexts and are characterised by different pedagogical orientations, and political, economic and organisation cultures [18]. Such contextual diversity defines the success of implementing new educational innovations such as standards for scaffolding [5]. Future research should examine the usability of the standards in other health sciences programmes operating in other geo-political and educational contexts.

## Conclusion

Although global educational innovations such as standards have the potential to improve the quality of health sciences education, they are not immune to multiple interpretations resulting from their social construction. In this pilot study, we adapted the principles of usability evaluation to describe the feasibility of standards for scaffolding in a pre-registration nursing programme in a low-resource context. The pilot served its purpose to reveal the context-based ‘truth’ regarding the fidelity of a health sciences programme evaluation on scaffolding, as well as identifying the ideal contextual conditions in which the standards for scaffolding health sciences programmes would work best. The evidence supported the feasibility and practicality of the standards in a pre-registration nursing programme operating in a low-resource setting. The standards were applicable and useful in directing, supporting and evaluating scaffolding practices across the selected programme. The identified usability flaws highlighted the need for further revisions of the standards, and to add relevant annotations as required. The study findings assisted in refining the standards and making evidence-based recommendations relevant to a nursing programme in low-resource settings. Future research focusing on the feasibility of the standards in other health sciences programmes and contexts is recommended.

### Electronic supplementary material

Below is the link to the electronic supplementary material.

Supplementary Material 1

Supplementary Material 2

## Data Availability

The standards used for the pilot and the reviewed standards are included as supplementary material for this published article. The datasets used and/or analysed during this study are available from the corresponding author upon request.
