# Supplementary material for: Usability of Standards for Scaffolding in a Health Sciences Programme: A feasibility Study
Source: BMC Nurs. 2024 May 7;23:309. doi: 10.1186/s12912-024-01975-0 (PMC11075252; doi:10.1186/s12912-024-01975-0)
Supplement: Supplementary file 1 — Supplementary Material 1 [file 12912_2024_1975_MOESM1_ESM.pdf]

ADDENDUM: FACULTY INTERVIEW GUIDE

1. Tell me about your experience with the use of the standards for scaffolding in your programme. *Probe as necessary.*
2. What worked well and what did you struggle with during the pilot testing of the standards for scaffolding? *Use necessary probes.*
   - What were the positives or negatives regarding the application of the standards to evaluate scaffolding in your programme?
   - How realistic or practical are the standards and criteria for scaffolding?
   - How easy or difficult was it to find evidence to support the existence of the standards scaffolding practices?
3. What observations did you make regarding the scaffolding practices in the programme? *Use necessary probes.*
4. What would you change about the standards if given an opportunity? *Use necessary probes.*
5. Any suggestions or comments on the standards and or the pilot exercise?
