# Supplementary material for: Usability of Standards for Scaffolding in a Health Sciences Programme: A feasibility Study
Source: BMC Nurs. 2024 May 7;23:309. doi: 10.1186/s12912-024-01975-0 (PMC11075252; doi:10.1186/s12912-024-01975-0)
Supplement: Supplementary file 2 — Supplementary Material 2 [file 12912_2024_1975_MOESM2_ESM.pdf]

Heuristics Checklist on Standards for Scaffolding (Author Generated)

Dear Expert

Once again, thank you so much for showing interest and sparing your valuable time to participate in this study. Please find attached the Standards for Scaffolding Health Sciences Programmes and the Piloting Programme's Self-Assessment Report for easy reference.

During your participation in this study, you will be expected to:

1. Read through the programme’s self-assessment report provided. The programme tested standards’ usability drawing evidence from multiple data sources.

   NB: The report presented as a table is a summary of findings on each standard and criteria plus evidence used to conclude the existence of the criteria. The self-assessment report is believed to have yielded data on how the programme interpreted, applied, adapted, and used the developed standards for scaffolding.
2. As one of the expert evaluators, you will either use the heuristics checklist provided as a word document (Annexure H) or complete the checklist online (using the REDCap link provided) to assess and interpret the usability of the standards based on your interpretation of the self-assessment report. The checklist structured as five questions will guide you through evaluating how the programme correctly interpreted, applied, effected, and adapted the standards to its context based on self-assessment report findings. Therefore, the heuristics checklist will guide you in supporting or refuting any standards, their structuring or wording. Besides, it is expected that you will examine the appropriateness and relevance of the evidence presented by the programme against the standards’ requirements.
3. The checklist require you to agree or disagree on the usability of each standard. You will be expected to use the information obtained from your examination of the self-assessment report and report to answer heuristics questions on how usable you perceive the standards are regarding their application in the health sciences programme. Your responses on grading the usability will be captured either as a Yes, No or Maybe for each of the heuristics (Yes = 2; Maybe = 1; No = 0). Space for

additional comments and/or suggestions for restructuring the standards is provided for each standard.

For more information and clarification regarding your task in this study, you are welcome to contact the principal investigator at belovedmas@gmail.com

**Heuristics**	**STANDARD 1: STRUCTURING AND SEQUENCING**<br><br>The curricular content, learning tasks, and learning environment must be structured to promote profession-specific knowledge building	**STANDARD 2: RESOURCES AND TOOLS**<br><br>Essential resources and tools must be available and accessible to support students' learning	**STANDARD 3: STRUCTURING THE PROGRAMME**<br><br>The curriculum composition must demonstrate the application of the underlying frameworks that support the structuring of learning outcomes	**STANDARD 4: INSTRUCTIONAL TECHNIQUES THAT SUPPORT DEEP LEARNING**<br>Educators must use relevant instructional scaffolding techniques to support individuals or groups of students in constructing knowledge.
1. The standard and accompanying criteria are **clearly written**	Yes No Maybe	Yes No Maybe	Yes No Maybe	Yes No Maybe
	Comments:	Comments:	Comments:	Comments:

Heuristics	**STANDARD 1: STRUCTURING AND SEQUENCING**<br><br>The curricular content, learning tasks, and learning environment must be structured to promote profession-specific knowledge building	**STANDARD 2: RESOURCES AND TOOLS**<br><br>Essential resources and tools must be available and accessible to support students' learning	**STANDARD 3: STRUCTURING THE PROGRAMME**<br><br>The curriculum composition must demonstrate the application of the underlying frameworks that support the structuring of learning outcomes	**STANDARD 4: INSTRUCTIONAL TECHNIQUES THAT SUPPORT DEEP LEARNING**<br>Educators must use relevant instructional scaffolding techniques to support individuals or groups of students in constructing knowledge.
2. The standards and accompanying criteria are **phrased using appropriate** health sciences terminology	Yes No Maybe	Yes No Maybe	Yes No Maybe	Yes No Maybe
	Comments:	Comments:	Comments:	Comments:

**Heuristics**	**STANDARD 1: STRUCTURING AND SEQUENCING**<br><br>The curricular content, learning tasks, and learning environment must be structured to promote profession-specific knowledge building	**STANDARD 2: RESOURCES AND TOOLS**<br><br>Essential resources and tools must be available and accessible to support students' learning	**STANDARD 3: STRUCTURING THE PROGRAMME**<br><br>The curriculum composition must demonstrate the application of the underlying frameworks that support the structuring of learning outcomes	**STANDARD 4: INSTRUCTIONAL TECHNIQUES THAT SUPPORT DEEP LEARNING**<br>Educators must use relevant instructional scaffolding techniques to support individuals or groups of students in constructing knowledge.
3. The self assessment findings are **supported by evidence** that matches the standards/criteria requirements	Yes No Maybe	Yes No Maybe	Yes No Maybe	Yes No Maybe
	Comments:	Comments:	Comments:	Comments:

Heuristics	**STANDARD 1: STRUCTURING AND SEQUENCING**<br><br>The curricular content, learning tasks, and learning environment must be structured to promote profession-specific knowledge building	**STANDARD 2: RESOURCES AND TOOLS**<br><br>Essential resources and tools must be available and accessible to support students' learning	**STANDARD 3: STRUCTURING THE PROGRAMME**<br><br>The curriculum composition must demonstrate the application of the underlying frameworks that support the structuring of learning outcomes	**STANDARD 4: INSTRUCTIONAL TECHNIQUES THAT SUPPORT DEEP LEARNING**<br>Educators must use relevant instructional scaffolding techniques to support individuals or groups of students in constructing knowledge.
4. The standards and accompanying criteria are **useful** for health sciences programmes	Yes No Maybe	Yes No Maybe	Yes No Maybe	Yes No Maybe
	Comments:	Comments:	Comments:	Comments:

Heuristics	**STANDARD 1: STRUCTURING AND SEQUENCING**<br><br>The curricular content, learning tasks, and learning environment must be structured to promote profession-specific knowledge building	**STANDARD 2: RESOURCES AND TOOLS**<br><br>Essential resources and tools must be available and accessible to support students' learning	**STANDARD 3: STRUCTURING THE PROGRAMME**<br><br>The curriculum composition must demonstrate the application of the underlying frameworks that support the structuring of learning outcomes	**STANDARD 4: INSTRUCTIONAL TECHNIQUES THAT SUPPORT DEEP LEARNING**<br>Educators must use relevant instructional scaffolding techniques to support individuals or groups of students in constructing knowledge.
5. The standards or accompanying criteria are **effective** in evaluating scaffolding	Yes No Maybe	Yes No Maybe	Yes No Maybe	Yes No Maybe
	Comments:	Comments:	Comments:	Comments:
